# Amputation of the Cervix Uteri

**Published:** 1861-11

**Authors:** J. Marion Sims

**Affiliations:** Surgeon to the Woman’s Hospital, New York


### AMPUTATION OF THE CERVIX UTERI.

By J. MARION SIMS, M. D.,

SURGEON TO THE WOMAN'S HOSPITAL, NEW YORK.

(*From the Transactions of the Medical Society of the State of New York.*)

Amputation of the cervix uteri has long been an established operation. Lisfranc frequently performed it for what he considered incipient carcinoma. Recent investigations in uterine pathology, however, have demonstrated the fact that his cases were not cancerous, but simply those of chronic inflammation with induration and hypertrophy of the cervix. At the present time, Huguier, of Paris, probably performing this operation oftener than any other surgeon, believes that most displacements of the uterus depend upon chronic enlargement of the cervix, and, laying aside all the local remedies usually employed in such cases, resorts at once to amputation, for which he claims an almost unvarying success.

This operation has been frequently performed by Dr. Emmet and myself, at the Woman's Hospital, sometimes by the knife, at others by scissors, and several times by the ecraseur. A few cases will illustrate our practice. Mrs. E., aged 32, the mother of two children, the youngest three years old, had a muco-purulent vaginal discharge with profuse and somewhat painful menstruation ever since her last confinement. Examination revealed great engorgement and hypertrophy of the cervix uteri with granular erosion. She was treated as an out-patient for several months without improvement, and was eventually admitted to the Woman's Hospital. She was treated here for four months without any marked benefit, and it was at length determined to amputate the cervix.

The os was split open laterally nearly to the insertion of the vagina. The tissue was of almost gristly hardness. Instead of amputating the cervix squarely, the anterior half of the cervix was first removed, cutting in the direction of the os internum, after which the posterior half was removed in like manner, cutting in the same direction, and leaving a conical excavation with the apex upwards, this being done to remove as much as possible of the indurated tissue. This mode of operation should not be imitated, however, for the cicatrization of the wound almost obliterated the cervical canal which is now very small, notwithstanding repeated incisions and other measures for dilatation. The os is a little round opening, hardly large enough to admit a No. 1 bougie, while to the touch the cervix seems to be sitting upon instead of projecting into the vagina.

To guard against the unfortunate result in this case, I adopted the rule of amputating the cervix at two different sittings; thus, splitting it from side to side as before, removing one half, and leaving the parts to cicatrize till the next menstrual period had passed, then removing the other half. I continued to follow this plan in general until an incident occurred in November, 1860, which entirely re-models the whole operation.

A lady from a distant State, aged forty-six, a widow for many years, and the mother in early life of two children, was the subject of procidentia uteri, of fifteen years' standing. She menstruated regularly, her general health was perfect, and she suffered only from the mechanical inconveniences of the displacement. The cervix was elongated, and there was a fibrous tumor as large as an English walnut, in the posterior wall of the uterus. The depth of the organ was three and three quarter inches. She had worn a Meigs' ring for several months with scarcely any relief, as the cervix protruded through and presented at the os externum, despite every mechanical agency. Her physician fully recognized all the difficulties of the case, and proposed an amputation of the cervix, but was unwilling to perform it himself, and sent her to the Woman's Hospital.

As she was in great haste to return home, I determined to remove the whole cervix at one operation. When she was fully under the influence of ether, Dr. Pratt, the House Physician, informed me that the ecraseur was broken, hence there was no alternative but to amputate by the cutting process. For this purpose I used scissors, expecting the hemorrhage to be less than by the knife. I removed about five-eighths of an inch of the cervix, cutting at right angles, and within half an inch of the vaginal insertion. On beginning the operation, I intended to leave the cut surface to heal by the granulating process, which usually takes five or six weeks, but while sponging the wound and waiting for the hemorrhage to cease, I discovered that the stump could be covered over with healthy vaginal tissue, in the same manner that a stump of an arm or a leg is covered with skin after amputation by the circular method. This was done by passing four sutures of silver wire through the anterior and posterior borders of the wound, which, when tightly drawn, brought its edges into apposition in a straight line across the middle of the stump, covering it completely, but leaving a small central opening for the os, just over the outlet of the vertical canal.

The operation was followed by no constitutional disturbance. The sutures were removed at the end of a week, the parts having healed entirely by the first intention.

Mrs. W. aged 34. Married at 19. Sterile. Was admitted to the Woman's Hospital in January, 1861. She had suffered for eleven years with menorrhagia, accompanied with severe pain in the back and loins, which ceased during a period of six years, then returned at intervals, and had continued for five months, without intermission, before her entrance into the hospital. She also had leucorrhœa whenever the hemorrhage ceased. The uterus was enlarged and somewhat retroverted. The cervix was elongated, hypertrophied and indurated, and the posterior lip was covered with florid vegetations which bled profusely on being touched. I at once proposed amputation, to which she readily consented. The operation was performed in the manner before described and with the same results. She now menstruates regularly, the flow lasting but three days, and is otherwise well.

Mrs. R., aged 38, the mother of two children, eighteen and sixteen respectively, and for five years a widow, was subject to leucorrhœa of ten years' standing. She consulted me in February, 1860. She had an enormous enlargement of the cervix uteri, with chronic inflammation, and a profuse discharge of an albumino-purulent character. She was treated by scarification, *potassa cum calce*, and other local means, for four months, with some improvement, but it seemed utterly impossible to effect a cure. I saw her in October and November, and again in January, and found her in about the same condition as on her first visit. I then suggested to her amputation as the speediest and best method of cure, but it was inconvenient for her to submit to it at that time, and the usual course of treatment, under such circumstances, was adopted. Her improvement was slow and unsatisfactory, and she at length insisted on having the operation performed, which was done April 29, 1861, in the presence of Dr. Parigot, of Brussels, Dr. Holcomb, of New York, and Dr. Pratt, of the Woman's Hospital.

The intra-vaginal portion of the cervix was nearly an inch in length and two inches in diameter. The anterior lip was a little larger than the posterior. The operation was performed as in the preceding cases, namely, by splitting the cervix laterally, and removing first, the anterior, and then the posterior half, by means of scissors. After the hemorrhage had ceased, the vaginal mucus membrane was drawn over the stump, as in the other cases, and secured by four silver sutures, two on each side of the cervical opening. There was no suppuration, no pain, no febrile excitement, and indeed the patient felt so well that, on the next day, she insisted on getting out of bed and going to the parlor; for prudential reasons, however, she was confined to her room a week. Here was a case which had been under treatment for fifteen months at various times, with little improvement, cured in a week, by this simple operation. The neck of the uterus now presents an appearance so normal that one could hardly believe that it had ever been the subject of disease or of a surgical operation.

In the old method of amputation, the suppuration continued for five or six weeks, and sometimes longer, before the parts were entirely cicatrized. According to this plan, there is no suppuration, the parts healing by the first intention and the patient becoming well in a week. In the former, there was danger of pyæmia; in this, there is little, if any; in the former, there was risk of degeneration in tissue—in this, there is none. In the former, the parts contracted as they healed; in this, they remain normal. It might have been supposed, *ápriori*, that this operation would have been attended with troublesome hemorrhage, but in each case it ceased as soon as the vaginal tissue was drawn over the stump, and in no instance was a ligature used. In all three of the cases, menstruation has been easy since the operation, though the os was smaller in each than natural, and required to be enlarged by sight incisions.

In amputating the tonsils, it is not necessary to cut out the entire gland; the removal of a section will suffice, as a large part of the remainder will disappear by absorption. In removing an intra-uterine polypus, it is not essential to cut the pedicle close to the wall of the uterus; if an inch of it is left it will disappear. So in amputating the cervix uteri, it is not necessary to cut it off even with the vaginal insertion to insure a good result, for whatever is left will be decreased by a modified nutrition, just as we see after amputation of the arm or leg.

